# Nonflammable Ether and Phosphate-Based Liquid Electrolytes for Sodium-Ion Batteries

**DOI:** 10.1021/acsami.4c11797

**Published:** 2024-10-03

**Authors:** Wessel W. A. van Ekeren, Alexandre M. Pereira, Marcelo Albuquerque, Luciano T. Costa, Reza Younesi

**Affiliations:** † Department of Chemistry-Ångström Laboratory, Uppsala University, SE-751 21 Uppsala, Sweden; ‡ MolMod-CS, Physical Chemistry Department, Institute of Chemistry, Fluminense Federal University, Campus Valonguinho, CEP 24020-141 Niterói, Rio de Janeiro, Brazil; § Institute of Physics, Fluminense Federal University, Campus Praia Vermelha, CEP 24210-346 Niterói, Rio de Janeiro, Brazil

**Keywords:** computational, galvanostatic cycling, NMR, nonflammable liquid electrolytes, sodium-ion batteries, solvation structure

## Abstract

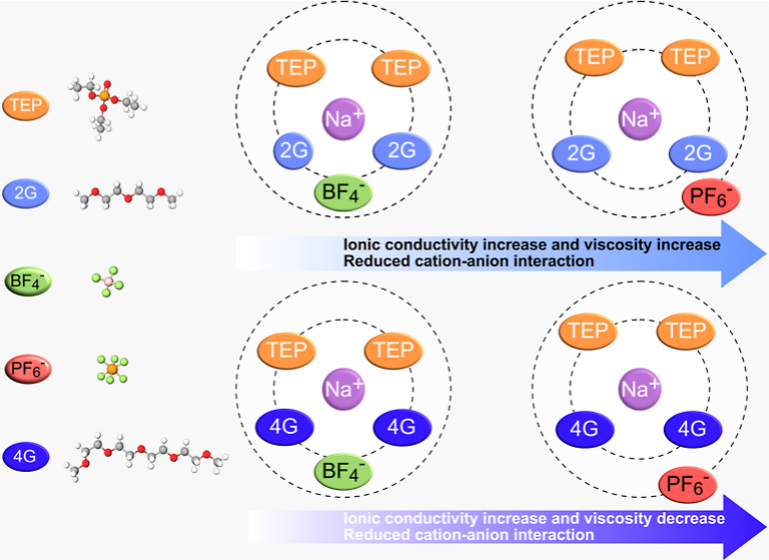

This study investigates a group of electrolytes containing NaPF_6_ or NaBF_4_ salts in phosphate- and ether-based solvents for high-mass loading sodium-ion batteries. It explores physicochemical properties such as ionic conductivity, dynamic viscosities, and nonflammability. The combination of experimental with computational studies reveals detailed insights into the physicochemical properties of the nonflammable liquid electrolytes. Diglyme-based electrolytes become nonflammable with 50 vol % phosphate solvents, while tetraglyme-based electrolytes require 70 vol %. The solvation structure has been investigated using NMR and is combined with computational studies to provide information about properties such as solvation structure, ionic conductivity, and viscosity. The molecular dynamic simulations confirm the enhanced solvation in diglyme-based liquid electrolytes observed experimentally by ^23^Na-NMR. Despite lacking sufficient electrochemical stability, this work provides a fundamental understanding of the solvation structure and physicochemical properties of a novel electrolyte system. This is an important contribution to be applied in future electrolyte design rationale.

## 1 Introduction

The interest in sodium-ion batteries is rapidly increasing worldwide, driven by their potential for large-scale energy storage and increasing relevance in the electric vehicle space.^1^ Because of the practically infinite availability of sodium from the ocean, sodium-ion batteries are very promising in terms of their low-cost and environmentally friendliness.^2^ However, although sodium-ion batteries are claimed to be safer than lithium–ion batteries due to their lower energy density,^3^ safety risks still exist because commercially used liquid electrolytes consist of flammable carbonate-based solvents. Among other strategies to address this concern, which are described in an earlier review paper by our group, nonflammable liquid electrolytes have recently received growing research interest.^4−6^ The implementation of nonflammable electrolytes is crucial for minimizing the risk of catastrophic failures, such as fires and explosions, without compromising electrochemical performance. This improvement is critical to making these batteries more practical and reliable for widespread use.

Fluorinated and alkyl phosphate-based solvents are the most well-known nonflammable solvents because of their hydrogen radical scavenging ability.^7^ However, fluorinated-based solvents are not environmentally friendly, and phosphate-based solvents do not form a stable solid electrolyte interphase (SEI) during the initial electrolyte reduction at the negative electrode. To overcome this issue, alkyl phosphate-based solvents could be optimized by using cosolvents, electrolyte additives, or high concentrations of salts.^8^ Here, a group of linear ethers and alkyl phosphates in different ratios are used as a binary solvent mixture, to which NaPF_6_ (conventional, but rather high-cost) or NaBF_4_ (less common, low-cost alternative) salts are added as the electrolyte salt. Ethers (glymes) are a promising group of electrolyte solvents for batteries because of their superior electrochemical performance, sufficient ionic conductivity, low viscosity, high stability at reducing potentials, and capability in the formation of efficient SEI with low resistance. However, they are not used at a commercial scale because of their toxicity.^9,10^ Especially bis(2-methoxyethyl) ether (diglyme, 2G) should be handled with care, which is labeled by the Environmental Protection Agency (EPA) as “high concern to workers, consumers, and children”.^11^ When handled with care, these glymes are still relevant for fundamental research purposes. The combination of ethers’ ability to form a stable SEI with low resistance and nonflammable alkyl-phosphate solvents allows for the utilization of the best properties of both solvent classes. The phosphate-based solvents used in this study are triethylphosphate (TEP) and trimethylphosphate (TMP), which are known to be nonflammable/flame retardant. To target enhanced ionic conductivity and electrochemical performance, these phosphates were mixed with 2G and tetraethylene glycol dimethyl ether (tetraglyme, 4G). For each electrolyte solvent mixture, five different volume ratios were analyzed (glyme/phosphate); 1:9, 3:7, 1:1, 7:3, and 9:1. Ideally, the phosphate content is as low as possible to minimize its poor SEI formation capability while maintaining the nonflammability. This work provides new insights toward the development of nonflammable liquid electrolytes for sodium-ion batteries in terms of both physicochemical properties and electrochemical performance.

## 2 Results and Discussion

### 2.1 Flammability

**Figure 1:**
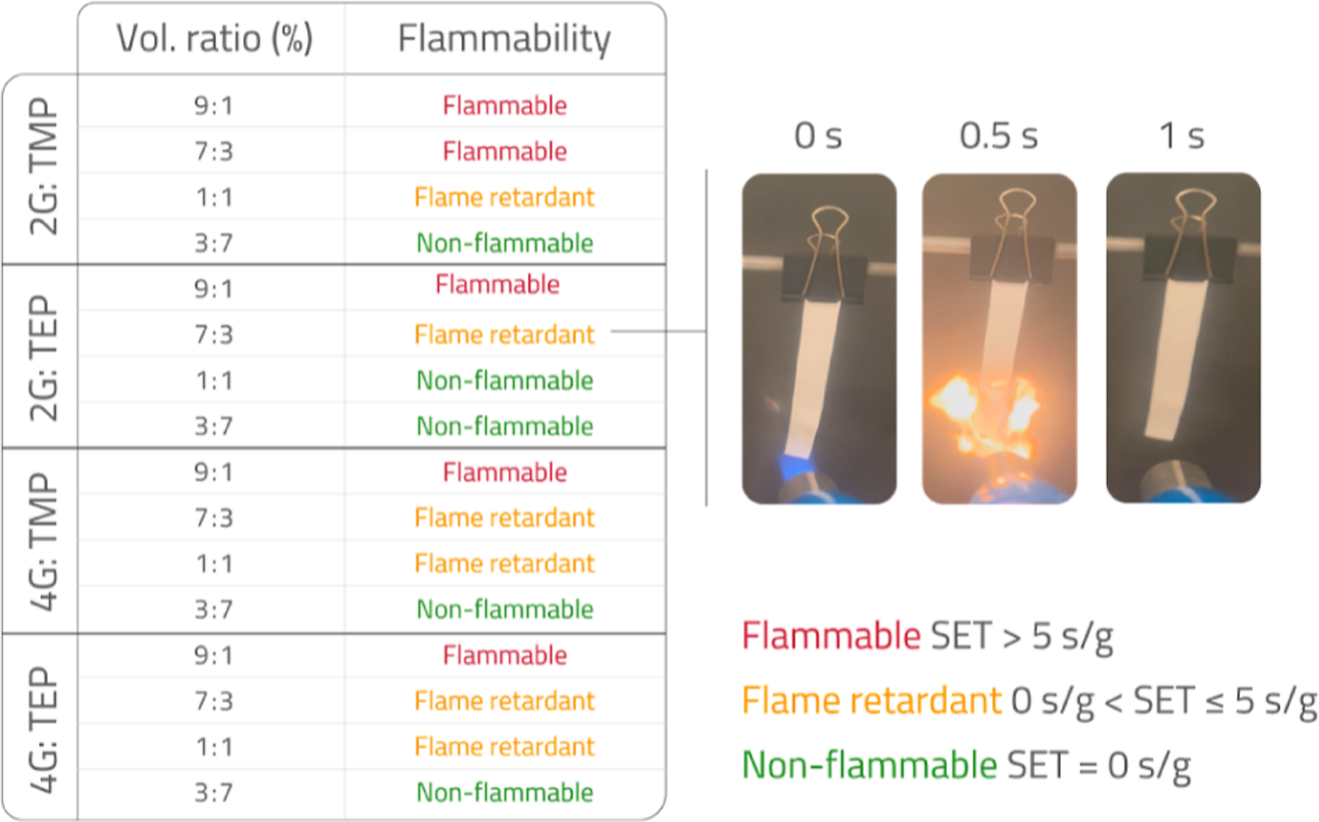
Overview of nonflammability characteristics for glyme and phosphate solvent mixtures.

The classification of flammability was based on the self-extinguishing time (SET, s/g), a method described in more detail elsewhere.^12^ However, in terms of nonflammability, we believe that the classification could be slightly stricter. The nonflammability results are classified as follows: (1) nonflammable when SET = 0 s/g, (2) flame-retardant when 0 s/g < SET < 5 s/g SET, and (3) flammable when SET > 5 s/g. In Figure 1, an overview of the nonflammability characteristics of the different solvent mixtures is shown.

Comparing the effects of TEP and TMP, it is clear that TEP has the strongest effect on nonflammability. The 2G/TEP mixtures show the best nonflammability characteristics. It should be noted that solely the solvent mixtures were tested without the addition of the salts. However, the addition of a noncombustible salt would most likely only enhance the nonflammability characteristics, due to the nonoxidizing nature of NaPF_6_ and NaBF_4_. The flammability results show that at least 70% TEP or TMP is required to make 4G nonflammable (the soaked glass fiber is not ignited after removal of the flame). This is in contrast with the work by Balaya et al.,^13^ where it was demonstrated that 1 M NaBF_4_ in 4G is a nonflammable electrolyte (tested by igniting electrolyte in a coin cell holder). Figure S3 in the Supporting Information shows that ∼1 M NaBF_4_ in 4G tested by this method is flammable (SET > 5 s/g). It should therefore be emphasized that the method of testing nonflammability is highly arbitrary and should be interpreted with care.

### 2.2 Relation between Viscosity and Ionic Conductivity

**Figure 2:**
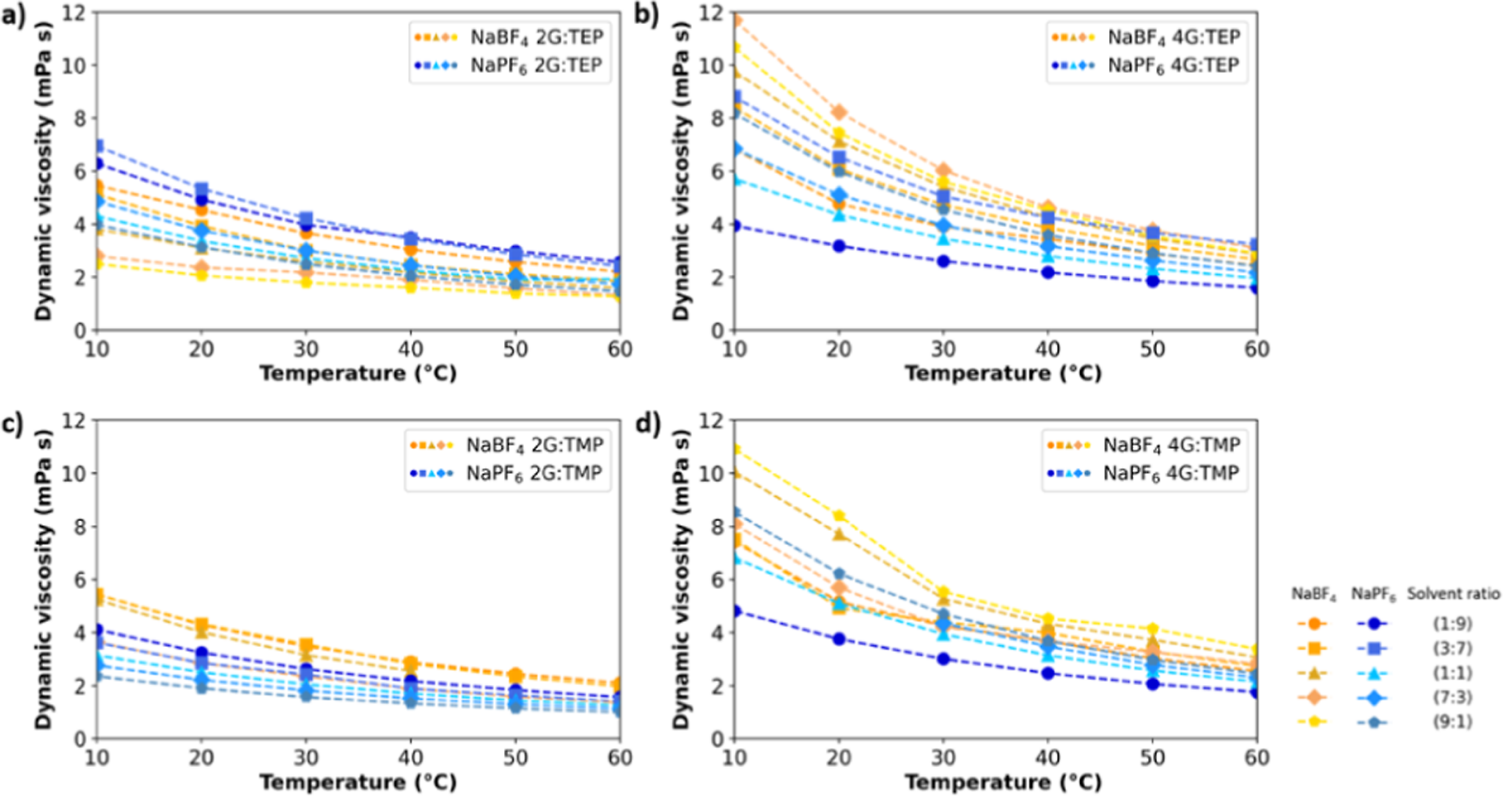
Dynamic viscosities for 1 m NaPF_6_ and 1 m NaBF_4_ in (a) 2G/TEP, (b) 4G/TEP, (c) 2G/TMP, and (d) 4G/TMP mixtures, in the temperature range of 10 to 60 °C.

The dynamic viscosity data is shown in Figure 2a–d for NaPF_6_- and NaBF_4_-based electrolytes, respectively, in the temperature range of 10–60 °C. There is no significant difference in terms of viscosity values if 1 molal of either NaPF_6_ or NaBF_4_ is dissolved in any of the solvent mixtures, i.e., all viscosity values are in the same order of magnitude. However, it should be noted that NaBF_4_ in the 2G/TEP mixture results in lower viscosity than the corresponding NaPF_6_-based electrolytes. This is a remarkable trend since, for all other solvent mixes, the NaBF_4_-based electrolyte results in the highest dynamic viscosity. The interplay between 2G and TEP can affect the dielectric properties of the electrolyte and most likely makes the dissociation of NaBF_4_ easier, compared to the dissociation in the other glyme and phosphate mixtures. Combining the Nernst–Einstein and Stokes–Einstein relations,^14^ which are only valid at infinite dilution, the relation between ionic conductivity (κ) and viscosity (η) can be described as
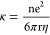
1where κ is the ionic conductivity, *n* is the number of charge carriers per unit volume, *e* is the elementary charge, *r* is the ionic radius, and η is the viscosity. The equation indicates that for electrolytes at infinite dilution, a lower viscosity results in higher ionic conductivities. However, the lower viscosity values of NaBF_4_ in 2G/TEP mixtures (about 4 mPa s at 20 °C) have not resulted in higher ionic conductivities than those for higher viscosity NaPF_6_ in 2G/TEP electrolytes (about 5 mPa s at 20 °C). This highlights that the simplistic relationship between viscosity and ionic conductivity does not always hold true in more complex systems of liquid electrolytes, where the effect of ion-pairing starts to play a more profound role. New theoretical models have been developed to enhance the understanding of the relationship between viscosity and ionic conductivity (transport properties) of more complex liquid electrolytes, mostly in aqueous systems.^15−17^

**Figure 3:**
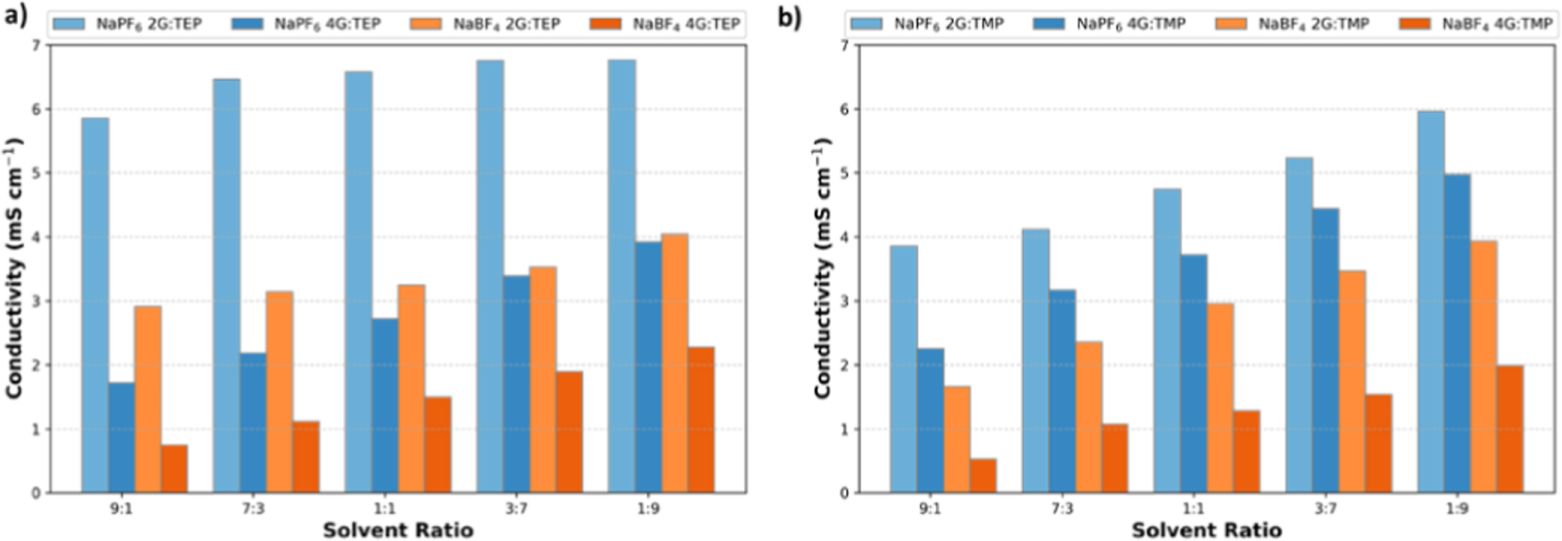
Ionic conductivities of electrolytes with 1 m NaPF_6_ and NaBF_4_ in solvent mixtures of (a) 2G or 4G with TEP and (b) 2G or 4G with TMP.

While these models and systems provide fundamental theories, they require further experimental validation, particularly in nonaqueous (aprotic) systems. The physicochemical properties studied here offer valuable experimental data for establishing a correlation between the viscosity and ionic conductivity in aprotic electrolytes. The ionic conductivities (κ), shown in Figure 3, of NaPF_6_ in 2G/TEP are noticeably higher than for NaBF_4_ in 2G/TEP, 6 mS cm^–1^ vs 3 mS cm^–1^, while the former possesses slightly higher viscosity (∼3–6 mPa s) compared to the latter (∼2–5 mPa s). One possible explanation for the enhanced ionic conductivities observed in NaPF_6_ compared to NaBF_4_ within this electrolyte solvent mixture could be attributed to the superior salt dissociation of NaPF_6_. As a result, the NaPF_6_-based electrolytes exhibit reduced ion-pairing, ultimately resulting in higher ionic conductivities. This effect is not present in the 4G and phosphate mixtures, where the viscosity values of NaBF_4_-based electrolytes are slightly higher (7 mPa s) than for NaPF_6_-based electrolytes (5 mPa s) and do explain the lower ionic conductivities of NaBF_4_ vs NaPF_6_ (2.5 mS cm^–1^ vs 4 mS cm^–1^). The cosolvation of 4G, a more viscous solvent on its own, results in a more viscous electrolyte solution compared to the use of 2G. This is not only due to the higher viscosity of 4G but also due to the solvation ability of this solvent. Tetraglyme provides more oxygen sites for the Na^+^ to electrostatically interact with, creating a stronger solvation and thus lower ionic conductivities.^18^ This finding is later in this work also confirmed by computational work. So, the dissociation ability of the salt in a certain solvent mixture is a prominent factor in relating the dynamic viscosity to the ionic conductivity.

### 2.3 Solvation Structure by ^23^Na-NMR and Computational Studies

**Figure 4:**
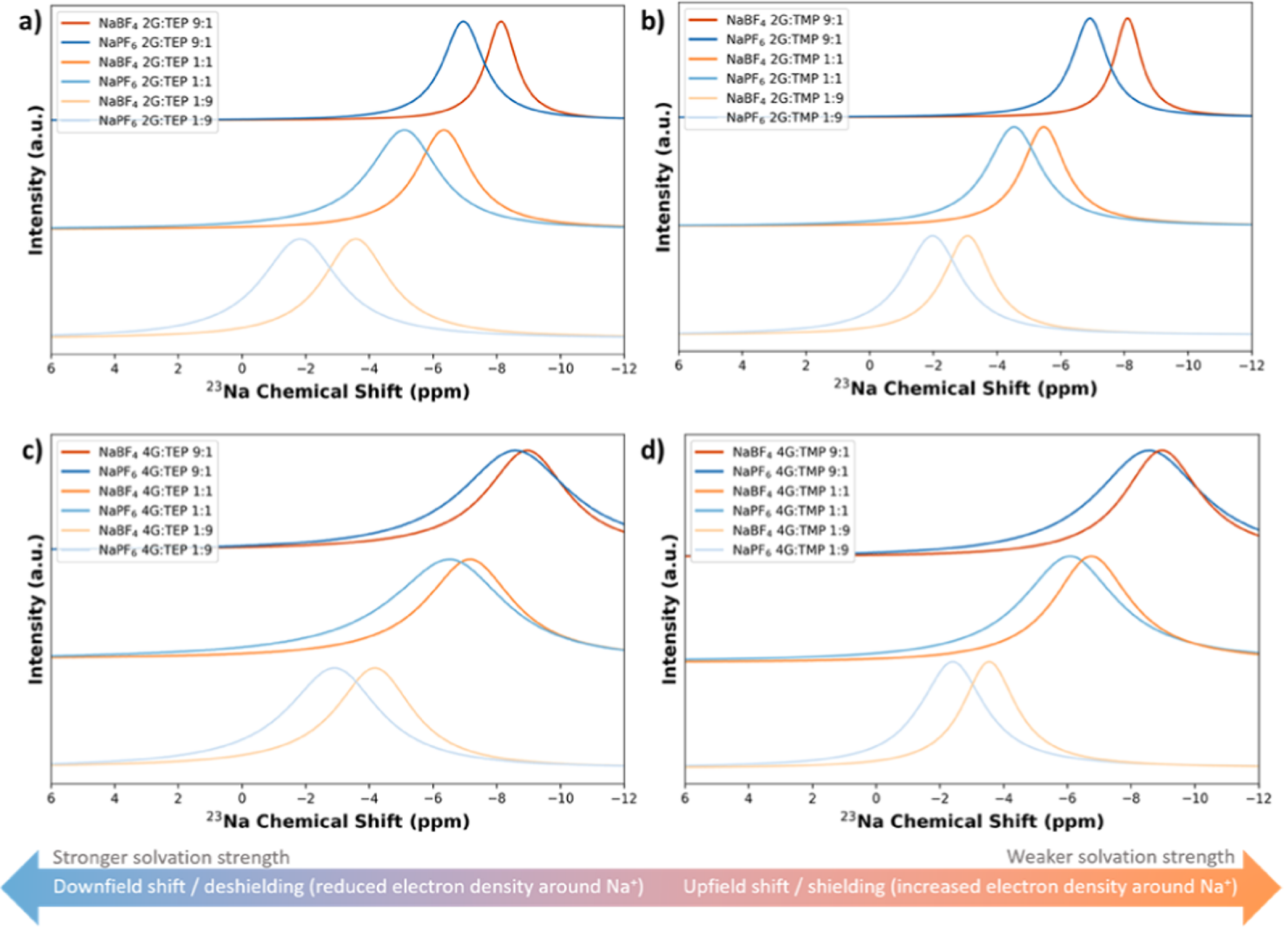
Change of coordination environment by ^23^Na NMR in electrolytes with different solvent mixtures (a) 2G/TEP, (b) 2G/TMP, (c) 4G/TEP, and (d) 4G/TMP.

To gain further understanding of the solvation environment and its relation to the ionic conductivity, nuclear magnetic resonance spectroscopy (NMR) and computational studies have been combined. In Figure 4, the ^23^Na-NMR results are shown for both NaPF_6_- and NaBF_4_-based salts in different solvent mixtures of glymes and phosphates (2G/TEP, 2G/TMP, 4G/TEP, and 4G/TMP). With an increase in the glyme/phosphate ratio (i.e., higher glyme content), the ^23^Na nucleus experiences a larger upfield shift. Generally, a more upfield chemical shift indicates a less electronegative environment around the Na nucleus.^19^ So, this upfield shift means that the Na nucleus in the high-content glyme mixture has a less electronegative environment compared to the low-content glyme mixtures. In the high-content glyme mixture, diglyme predominantly dominates in the coordination of Na, leading to more 2G[O]–Na interactions. The observed upfield chemical shift in the ^23^Na NMR spectra of glyme-rich electrolytes can be explained by the computed electronegativity (χ) values. χ of the respective solvent has been calculated using Koopmans’ theorem, where the ionization energy (*I*) is equivalent to the negative value of the highest occupied molecular orbital (HOMO) energy and the electron affinity (*A*) is equivalent to the negative value of the lowest unoccupied molecular orbital (LUMO) energy. The calculated HOMO and LUMO values are shown in Figure S5. To compute the electronegativity, the relation (*I* + *A*)/2 has been applied, where *I* and *A* are, respectively, the ionization potential and electron affinity.^20^ It was found that the phosphates have an electronegativity of approximately 3.5 and that glymes have an electronegativity of around 3.0, as shown in Table S3. Therefore, a higher glyme content results in a less electronegative environment of the Na^+^, causing the observed upfield shift. Conversely, a decrease in glyme content (i.e., higher phosphate content) results in a downfield shift caused by a more electronegative environment of the Na nucleus. Furthermore, the ^23^Na in the NaPF_6_-based electrolyte experiences a stronger downfield shift (deshielding) compared to NaBF_4_-based electrolytes, which means that Na^+^ in NaPF_6_-based electrolytes experiences a more electronegative environment compared to Na^+^ in NaBF_4_. This more electronegative environment around Na^+^ increases the solvation strength and thus the ion mobility. Lastly, it should be noted that more peak broadening is observed for the electrolytes with tetraglyme, which agrees with the higher viscosity values for these systems. The higher viscosity of tetraglyme will slow down the random rotational motion of the molecules (molecular tumbling) and thus results into peak broadening.^21^

**Figure 5:**
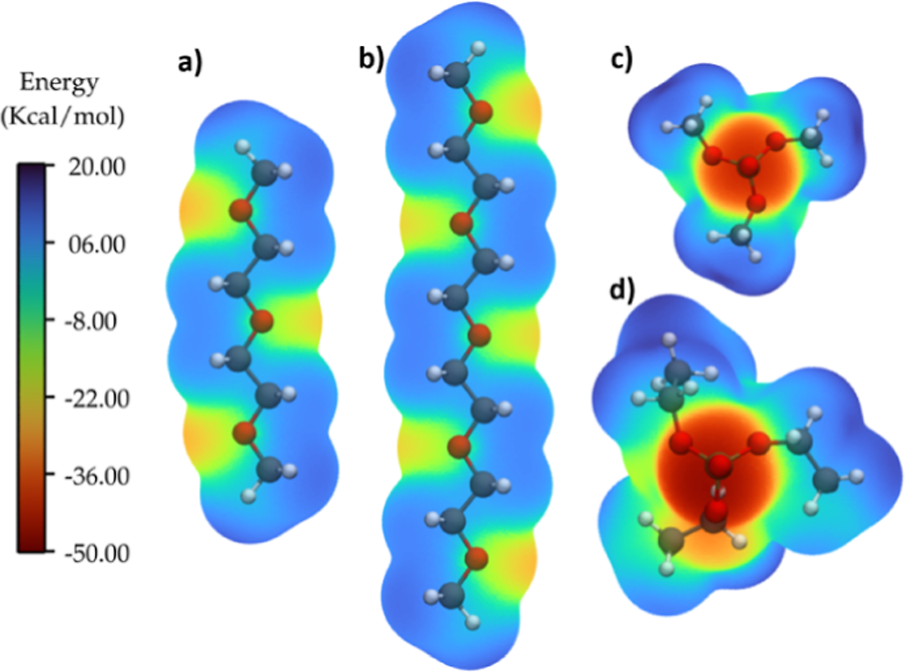
ESP maps of the glymes (a) 2G and (b) 4G and phosphates (c) TMP and (d) TEP.

The observed chemical shifts in the ^23^Na NMR spectra highlight changes in the solvation environment upon changes in the sodium salt and phosphate content. However, the specific interactions and preferred solvation of Na^+^ with certain solvent molecules or anions remain unclear. To enhance our understanding of the solvation structure, computational studies were conducted to obtain quantitative electrostatic surface potentials (ESP) using Multiwfn and radial distribution functions (RDF).^22,23^ These studies provide insights into the specific interactions between Na^+^ and O atoms of the phosphate and glyme molecules. From Figure 5, in which the obtained ESP is shown, the expected electrostatic interactions between Na and the specific atoms in the solvent molecules can be determined. The phosphate (=O) sites exhibit higher electrostatic potential compared to the glyme oxygens, which have approximately half the energy of the phosphate groups. The flexibility of glyme molecules allows multiple oxygen sites to interact with Na^+^ (2G has three interaction sites and 4G has five interaction sites), whereas in the phosphate molecules, being less flexible, only one oxygen site interacts with Na^+^. So, in phosphate-rich solvent mixtures, more phosphate molecules are required to complete the solvation sheath around Na^+^. Assuming that every electrophilic site in the molecule interacts with an ion, 4G exhibits the strongest interaction, followed by 2G, TEP, and TMP.

**Figure 6:**
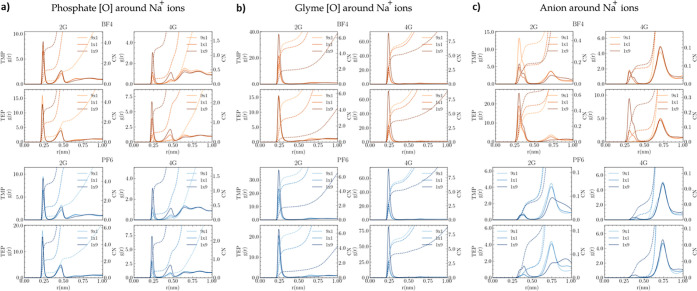
RDF, *g*(*r*), and the coordination numbers (CN, dashed lines) of (a) phosphate oxygen atoms around Na^+^ ions, (b) glyme oxygen atoms around Na^+^ ions, and (c) BF_4_^–^–Na^+^ interactions and PF_6_^–^–Na^+^ interactions using volume ratios of glyme/phosphate of 9:1, 1:1, and 1:9 at 293 K.

The RDFs, (*g*(*r*)), were calculated for the interactions between Na^+^ ions and the oxygen atoms in phosphate and glymes, as shown in Figure 6a,b. Similar trends were observed for all of the O–Na^+^ interactions: glyme oxygens exhibited higher peaks in *g*(*r*), indicating stronger interactions compared to phosphate oxygens, consistent with the ESP results. The 4G exhibits the strongest interactions with Na^+^ ions, showing peaks higher in intensity than those of phosphate oxygens. Despite this dominance, phosphates were still present in the same solvation sheath, albeit with lower occurrence. The *g*(*r*) profiles and intensities for phosphate[O]–Na^+^ interactions were similar in both PF_6_^–^- and BF_4_^–^-based systems. However, the strongest effect is observed when changing the ether length, comparing 2G and 4G. In 2G systems, the first peak was approximately twice as high, indicating more frequent phosphate–Na interactions compared to 4G systems. The highest coordination number (CN) (dashed lines in Figure 6) observed was 5 in a 2G phosphate system with PF_6_^–^ (see Figure 6a), suggesting this may be the maximum number of phosphate molecules that can arrange around Na^+^ ions without steric hindrance. However, in the 4G/phosphate-based electrolytes, the effect of steric hindrance appears earlier, and a maximum CN of 2 phosphate molecules around Na^+^ is found for the system with TMP and PF_6_^–^, as shown in Figure 6a.

The *g*(*r*) for glyme[O]–Na^+^ interactions, shown in Figure 6b, has more intense peaks compared to phosphate[O]–Na^+^, indicating the highest energy interactions due to their multiple interacting sites and the ability to “embrace” Na^+^ ions. The solvation structure of the 4G/phosphate-based electrolytes remains similar for TMP and TEP. Although 2G shows lower intensity than 4G, it is still a rather strong interaction compared to that of the phosphate[O]–Na^+^. Also, for the 2G/phosphate-based electrolytes, TEP seems to be competing more strongly with 2G compared to TMP. In the 2G/TEP-based electrolytes, 2G is less likely to be interacting with Na^+^ ions compared to the 2G/TMP-based electrolytes. The CNs for 4G[O]–Na^+^ interactions exceed 6, demonstrating the molecule’s ability to coordinate around Na^+^ ions using multiple sites simultaneously. The expected interaction order from ESP maps is also reflected in *g*(*r*): 4G > 2G > TEP > TMP. However, in the 2G/TEP system, TEP–Na^+^ interactions are higher than those of 2G–Na^+^ interactions. This suggests a synergistic interaction between BF_4_^–^ and TEP that may suppress the 2G interaction, as illustrated in Figure S4. This trend is well correlated with the high viscosity presented for the systems containing BF_4_^–^ ions, as shown in Figure 2.

From the *g*(*r*) of the interaction between the anion and cation in Figure 6c, it can be clearly observed that BF_4_^–^ participates in the first solvation sheath, whereas PF_6_^–^ participates more in the second solvation sheath. In the NaPF_6_-based electrolytes, the larger volume of PF_6_^–^ hinders stronger interactions between the anion and the cation. Notably, in the 2G/TEP solvent mixture, PF_6_^–^ shows a higher probability of being in the first solvation sheath, though this tendency is less pronounced compared to BF_4_^–^. When BF_4_^–^ ions are present in the first solvation sheath, this leads to the formation of contact ion pairs, which can explain the earlier discussed lower ionic conductivities for NaBF_4_-based electrolytes. This observation also aligns with the NMR results, where the ^23^Na chemical shifts experience a stronger upfield shift in the electrolytes based on NaBF_4_, compared to that for NaPF_6_. This suggests that in the NaBF_4_-based electrolyte more Na^+^ ions are shielded, which is consistent with the peak displacements in *g*(*r*). Comparing the effect of chain length (2G versus 4G), it appears that the ethers with shorter chain length (2G) have a lower probability of being close to Na^+^ compared to 4G. In other words, there are more phosphate and anions close to Na^+^ in the first solvation sheath in the system with NaBF_4_ salt.

### 2.4 Electrochemistry in High-Mass Loading Prussian White|Hard Carbon Full-Cells

**Figure 7:**
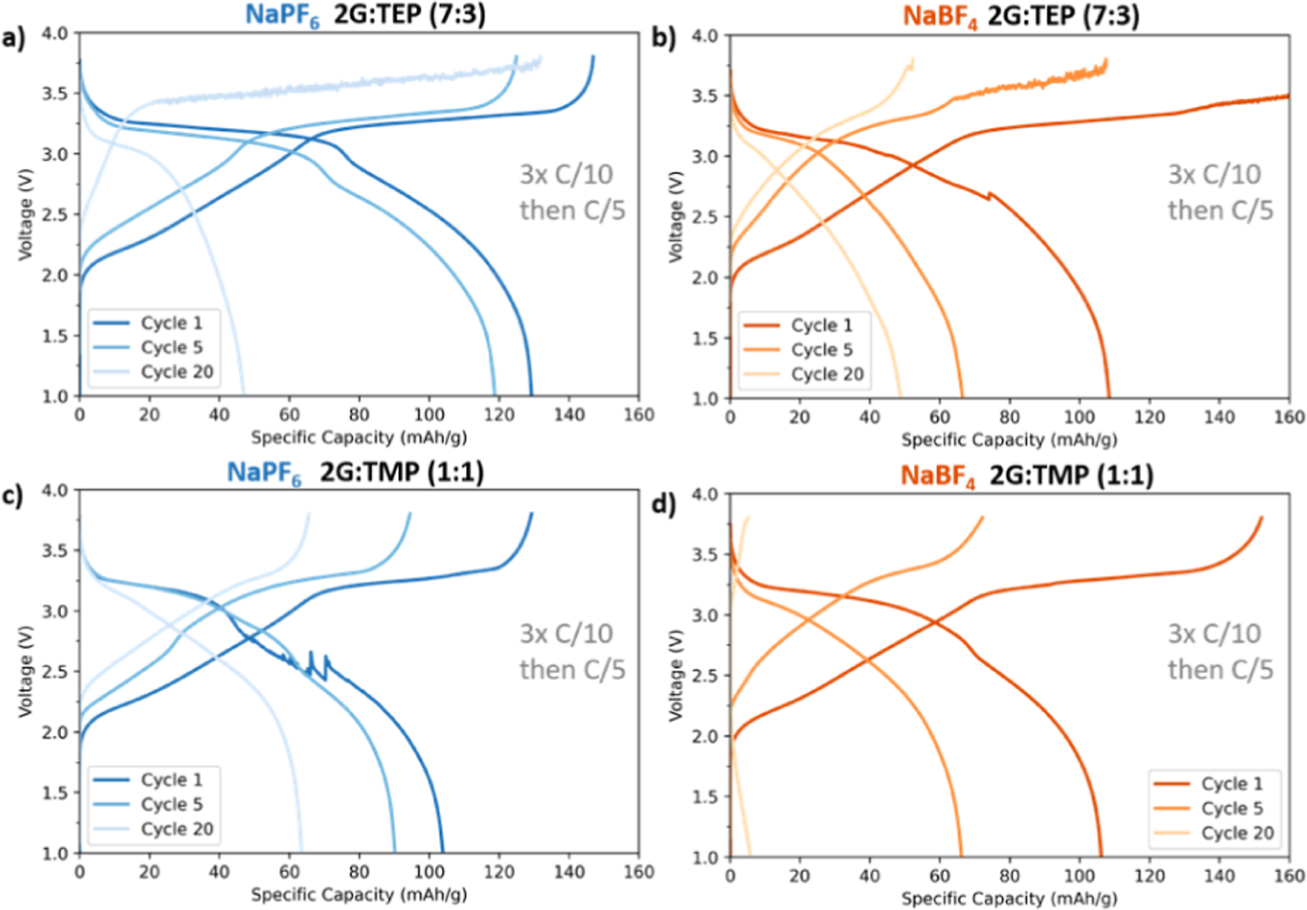
Discharge capacities in high-mass loading PW|HC full-cells for (a) 1 m NaPF_6_ in 2G/TEP (7:3), (b) 1 m NaBF_4_ in 2G/TEP (7:3), (c) 1 m NaPF_6_ in 2G/TMP (1:1), and (d) 1 m NaBF_4_ in 2G/TMP (1:1).

**Figure 8:**
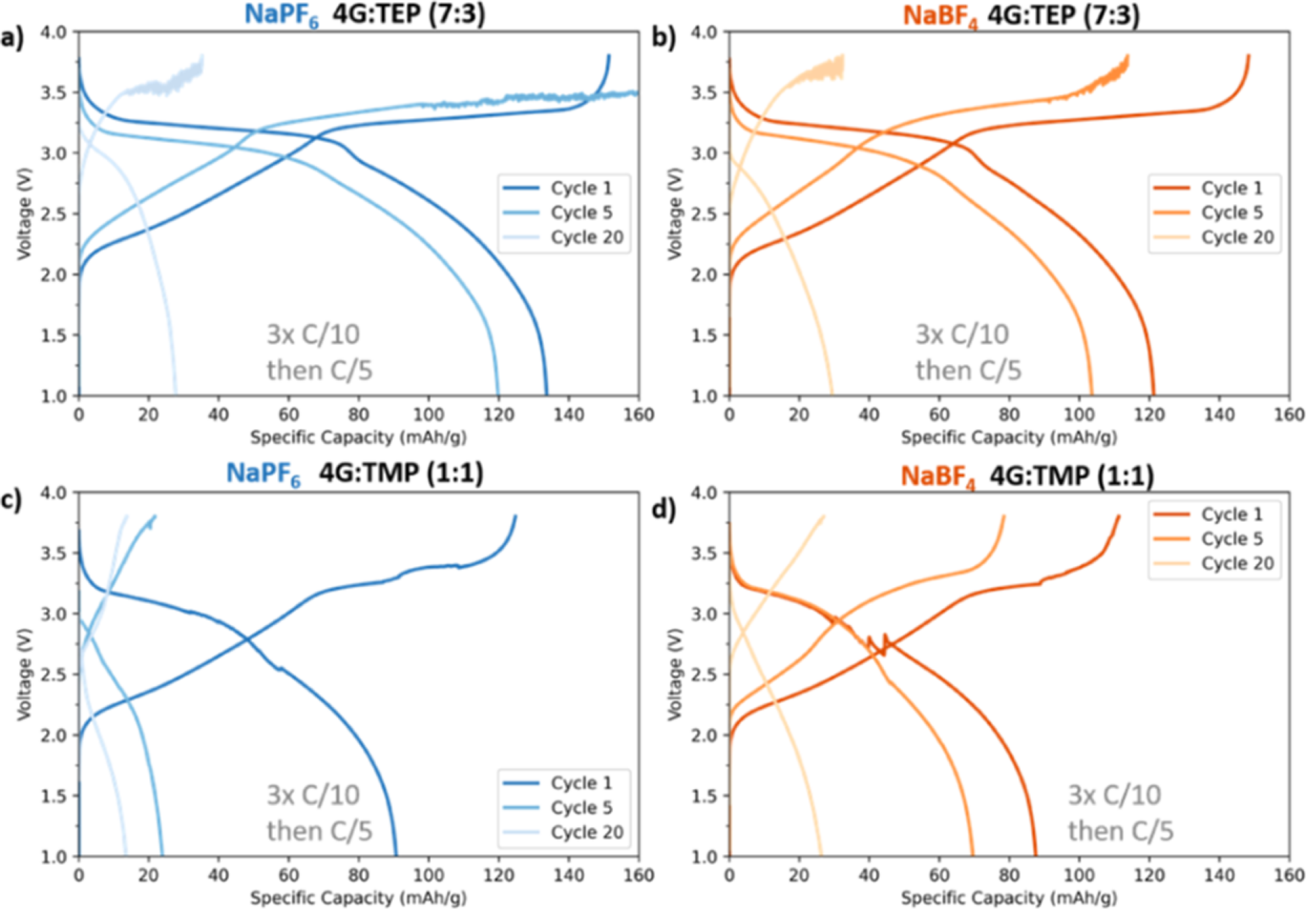
Discharge capacities in high-mass loading PW|HC full-cells for (a) 1 m NaPF_6_ in 4G/TEP (7:3), (b) 1 m NaBF_4_ in 4G/TEP (7:3), (c) 1 m NaPF_6_ in 4G/TMP (7:3), and (d) 1 m NaBF_4_ in 4G/TMP (7:3).

The aforementioned electrolytes were tested in full-cell sodium-ion batteries using Prussian white (PW, 12 mg/cm^2^) vs hard carbon (HC, 7 mg/cm^2^) electrodes. The selection of solvent mixture to continue testing in galvanostatic cycling was based on the requirement of being at least flame-retardant and having the highest content of glymes because of the known poor compatibility of phosphates with carbonaceous anodes. The galvanostatic discharge capacity curves are shown in Figures 7 and 8. The results indicate that NaPF_6_-based electrolytes show higher initial stability in most cases but still suffer from rapid capacity fades. The capacity fade is even more pronounced when a NaBF_4_ salt is used. The rather low initial Coulombic efficiency for all the tested electrolytes indicates poor SEI formation. The results also reveal that the overpotential increases from the first to the fifth cycle for all of the investigated electrolytes. This is more pronounced for the electrolytes based on the NaBF_4_ salt, suggesting that the resistance is higher in these cells. Also, the cells containing 4G solvent showed instability at the end of charge. This is most likely due to the plating of sodium metal due to the high viscosity and rather low ionic conductivity of the 4G-containing electrolytes.

Overall, given that glymes are known to form an efficient SEI, the poor SEI formation is primarily governed by the phosphate solvents. This could be explained by the fact that phosphate undergoes reduction at higher potentials before the reduction of glymes. Therefore, the SEI formed with these electrolytes is most likely phosphate-rich, thick, and unstable. To enhance the long-term stability of TEP-based solvent, rather than solely cosolvation with glymes, the addition of SEI forming additives such as vinylene carbonate (VC) or 1,3-propene sultone (PES) could be further explored or optimizing the SEI formation protocol to favor certain reduction processes.^24^

#### 2.4.1 *Operando* Pressure Evolution in High Mass-Loading PW|HC Full-Cells

**Figure 9:**
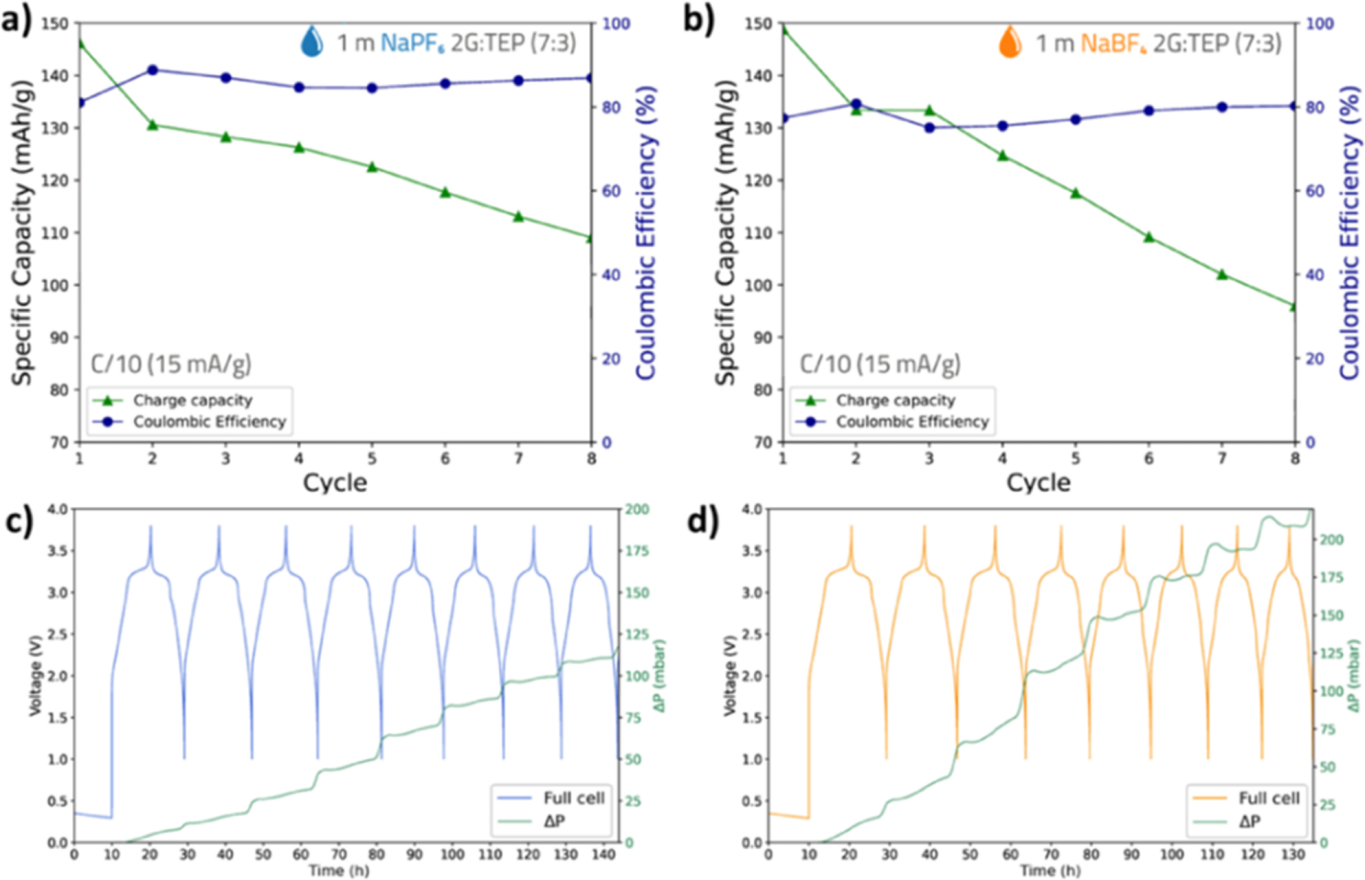
(a,b) Show the Coulombic efficiency and charge/discharge capacities in NaPF_6_- and NaBF_4_-based electrolytes, respectively. In (c,d), the charging profile is plotted together with the pressure evolution for NaPF_6_ and NaBF_4_, respectively.

To further elucidate the unstable cycling behavior using the solvent mixture of glymes and phosphates, we performed pressure evolution studies. The results are shown in Figure 9. It should be noted that the discharge capacities observed in the pressure cells are slightly higher than those in the pouch-cells due to cycling at a C-rate of C/10 and a constant temperature of 30 °C. There is a clear trend in pressure increase upon full discharge, where the pressure evolution rate is significantly higher compared to other states of charge. In the fully discharged state, the PW is fully sodiated, and the HC is fully desodiated, which are both associated with a volume decrease of the electrodes.^25,26^ Therefore, the pressure increase observed in the fully discharged state, around 1.0 V, can be fully attributed to the oxidation/decomposition of the electrolyte, resulting in the formation of gaseous products.

Overall, the pressure evolution clearly shows a notable increase when this solvent mixture is used during the formation cycling, with approximately a 25 mbar pressure increase observed with NaPF_6_ and a 75 mbar pressure increase with NaBF_4_. This is significantly different compared to our earlier study on the same PW|HC full-cell chemistry, but with NaBOB TEP-based electrolytes, where less than 6 mbar of pressure increase was observed during the formation cycling.^8^ In that study, a slightly higher C-rate was used for the formation cycles, which provided less time for each reaction to occur, resulting in lower amounts of gas evolution. Another factor contributing to reduced pressure evolution was the formation of a passivation layer due to the additives and different salts used, which reduced the amount of continuous electrolyte decomposition. The addition of glymes to the solvent mixture appeared to increase gas evolution compared with the earlier study without glymes. Moreover, gas evolution was more pronounced with the NaBF_4_-based electrolyte compared to the NaPF_6_-based electrolyte, which may explain the lower discharge capacities and greater capacity fade observed with this electrolyte.

## 3 Conclusions

In summary, the combination of glymes and phosphates is an interesting approach to formulating nonflammable electrolytes with relatively high ionic conductivities. Although the viscosity values of NaPF_6_- and NaBF_4_-based electrolytes were comparable, the NaPF_6_-based electrolytes exhibited remarkably higher ionic conductivities compared to NaBF_4_-based electrolytes. This highlights the critical role of the solvent in salt dissociation, where despite the high viscosity of the electrolyte solution, still high-ionic conductivities can be obtained. Among the investigated solvents, 2G/TEP and 2G/TMP solvent mixtures achieved the highest ionic conductivities compared with 4G-containing electrolytes, revealing the detrimental effect of the presence of 4G on the ionic conductivities. The ^23^Na NMR and computational studies demonstrated that higher glyme content leads to a less electronegative environment around the sodium ions, whereas a higher phosphate content and the presence of NaPF_6_ result in a more electronegative environment, thereby affecting the solvation strength. The ^23^Na NMR and computational analysis demonstrate that glyme-rich electrolytes create a less electronegative environment around Na^+^, reflected by an upfield shift in the ^23^Na NMR spectra. Moreover, NaBF_4_-based electrolytes form contact ion pairs in the first solvation sheath, resulting in lower ionic conductivities compared to NaPF_6_ electrolytes. Overall, the glyme length (2G vs 4G) and the type of phosphate (TEP vs TMP) significantly influence the solvation environment and resulting ionic conductivity, with longer glymes (4G) and weaker competing phosphates (TMP) generally providing the most favorable conditions for ionic transport. The electrochemical results indicate that the presence of phosphates, even with the lowest phosphate concentrations tested here, still faces challenges in cycling stability. Neither 1.0 m NaPF_6_ nor 1.0 m NaBF_4_-based electrolytes using a solvent mixture of glymes and phosphates showed promising cycling stability in high mass-loading PW|HC full-cells, except for NaPF_6_ in 2G/TEP (7:3). This work underscores that the use of phosphates in nonflammable liquid electrolytes with carbonaceous anodes requires further optimizations, either through the addition of higher salt concentrations, SEI forming electrolyte additives, or by optimizing the SEI formation protocol. By understanding the fundamentals of the solvation behavior of Na^+^, the physicochemical and electrochemical properties of liquid electrolytes can be further improved, paving the way for future electrolyte formulations.

## 4 Experimental Section

### 4.1 Preparation of Electrolytes

Sodium hexafluorophosphate (NaPF6, Fluorochem) and sodium tetrafluoroborate (NaBF4, Sigma-Aldrich) were dried in a vacuum oven at 120 °C for 12 h. Prior to use, the solvents TEP (Sigma-Aldrich, ≥99.8%), TMP (Sigma-Aldrich), bis(2-methoxyethyl) ether (diglyme, Sigma-Aldrich), and tetra(ethylene glycol) dimethyl ether (tetraglyme, Sigma-Aldrich) were dried over dehydrated molecular sieves (4.0 Å) for at least 48 h and filtered before use. Each electrolyte solution was prepared to obtain a molarity of 1.0 mol/g. All electrolyte mixing was done in an argon-filled glovebox (O_2_ < 1 ppm and H_2_O < 1 ppm). The electrolyte mixtures were stirred for 24 h or until clear solutions were obtained.

### 4.2 Physicochemical Properties

The viscosity and density of the electrolytes were analyzed using a Lovis 2000 M/ME (Anton Paar) operating between 10 and 60 °C. An overview of the data at 20 °C is shown in Table S2 and Figure S2. The conductivity measurements were carried out in the glovebox at room temperature (∼25 °C) using a Mettler Toledo SevenGo Duo pro pH/ORP/Ion/Conductivity meter SG78 instrument with an InLab 738ISM probe.

Flammability tests were performed by placing a few drops of electrolyte on 10 cm × 1 cm strips, which were subsequently exposed to a butane flame. All flammability tests were repeated twice, and the burning time was averaged. The results of the flammability test for 1.0 m NaBF4 in 4G are shown in Figure S3, which has previously been claimed to be nonflammable but is flammable using this method. This highlights the importance of the test method and how to interpret the flammability results of liquid electrolytes.

The NMR-measurements have been performed on a JEOL spectrometer (400 MHz), where chemical shifts were recorded in parts per million. The NMR samples were prepared in coaxial tubes, where the inner tube contained a deuterated reference solution (60 μL) and the outer tube contained the sample (560 μL). The 1H and 13C NMR signals were referenced to the signals of DMSO-*d*_6_ at 2.46 and 39.6 ppm, respectively. The ^23^Na signals were referenced to the signal of 0.1 M NaCl in D2O at 0 ppm.

### 4.3 Computational Studies

The density functional theory approach has been used to obtain the electron density and ESP maps for the TMP, TEP, 2G, and 4G structures.^22^ Optimized geometries, vibrational frequencies, and charge distributions were obtained using the ORCA 5.0.3 program package^27^ at the B3LYP^28,29^ hybrid functional and def2-TZVP^30^ as the basis set. The optimized wave function was the input for the postprocessing analysis using the Multiwfn package^23^ to obtain the restrained electrostatic potential (RESP)-derived charges and ESP maps. RESP charges were used for each atom type, which are defined in the Gromacs topology for the molecular dynamics (MD) simulations.

MD simulations were used to characterize the structural and dynamical properties of the nonflammable electrolytes proposed in the experimental section. The approach used for the MD simulations in this work follows the same procedure as published in an earlier work.^31^ For the force field nonbonded topologies, the fftool developed by Agilio Padua and co-workers^32^ has been used, with the same OPLS^33^ force field parameters for all the organic compounds. The force field parameters for the Na^+^, BF_4_^–^, and PF_6_^–^ ions were based on Acevedo’s work.^34^ For that, as shown in Figure S2, the charges were scaled by 0.8.

The investigated systems were packed in a cubic box using the PACKMOL^35^ package, where each system presents two ions (BF_4_^–^ or PF_6_^–^) with various compositions of mixtures containing TMP or TEP and either 2G or 4G. Temperatures ranging from 283.15 to 373.15 K were investigated. However, for the structural analysis, the results are shown at 293 K. A total of 500 solvent molecules were used, distributed in the following proportions: 1:1 (250 molecules each) and 1:9 (50:450 molecules). The number of Na^+^ ions varied from 32 to 54, depending on the density of the systems, with anions added until reaching zero charge, see Table S4. Topologies and additional information can also be found on GitHub link (www.github.com/molmodcs).

The GROMACS 2023.3^36^ package has been used for all of the MD simulations. Energy minimization was performed via the conjugate gradient algorithm, with a steepest descent step executed every ten steps. This was followed by 20 ns of stochastical dynamics simulation at 1.0 bar and the target temperature, utilizing a Berendsen barostat^37^ and v-rescale,^38^ with a coupling time of 1.0 ps. Subsequently, a 40 ns production run was carried out, employing C-rescale^39^ and v-rescale algorithms to regulate pressure and temperature, with coupling times set to 1.0 and 0.1 ps, respectively. Bonds containing hydrogens were constrained using the LINCS algorithm,^40^ and particle mesh Ewald^41^ was employed for Coulomb interactions. A 1.2 nm cutoff was applied for all interactions, with the Lennard–Jones potential smoothly switched off between 1.0 and 1.2 nm. Analysis was performed using GROMACS tools and TRAVIS.^42^ Additionally, we used the Visual Molecular Dynamics (VMD)^43^ tool to visualize the results.

### 4.4 Electrochemical Measurements

The electrolytes were tested in full-cell pouch configurations. Pouch cells were assembled using 18 mm PW, 21 mm HC electrodes, 2.5 × 2.5 cm Dreamweaver separator, and 100 μL of electrolyte in an argon-filled glovebox. Prior to use PW and HC were dried for 15 h under vacuum at 150 and 170 °C, respectively. The Dreamweaver Gold separator was dried at 150 °C for 15 h. The galvanostatic cycling tests were performed on a Neware BTS-4008-5V20mA battery tester at room temperature (around 20 °C). The cells were kept at the OCV for 12 h prior to cycling to ensure proper wetting of the electrodes. All pouch cells were cycled according to three formation cycles at C/10 (15 mA/g) and subsequently cycled at C/5 (30 mA/g) in the voltage range of 1.0–3.8 V. The C-rates are based on the practical specific capacity of PW, 150 mA h/g.

The operando electrochemical pressure measurements were carried out using a helium-leak tested pressure cell (PAT-Cell-Press) of El-CellGmbH and a Biologic potentiostat. The PAT-Cell-Press consists of a lower plunger, upper plunger, and insulation sleeve, which were all used as delivered by El-Cell. The plungers are made of aluminum, acting as current collectors. The insulation sleeve contained a predried borosilicate-glass fiber separator (Whatman, grade GF/A, 18 mm diameter, 260 μm). The cell setup was helium-leak tested and guaranteed a maximum leakage rate of 0.3 mbar h^–1^. The cells were assembled with 100 mL of electrolyte in an argon-filled glovebox (O_2_ < 1 ppm and H_2_O < 1 ppm). A slight variation in initial stack pressure of the cell might be observed because the upper lid of the cell is closed manually, but the OCV of 12 h ensured stable pressure prior to cycling. After assembly, the cells were placed in a climate chamber (KB53, Binder (KGmbH)) and cycled at 30 °C. The pressure cells were cycled at a C-rate of C/10 to give more insights in the gas formation, which is expected to be slightly higher at lower C-rates compared to higher C-rates.

## Supporting Information Available

Experimental section and computational methods (PDF)
