# Supplementary material for: Nonflammable Ether and Phosphate-Based Liquid Electrolytes for Sodium-Ion Batteries
Source: ACS Appl Mater Interfaces. 2024 Oct 3;16(41):56355–65. doi: 10.1021/acsami.4c11797 (PMC11492165; doi:10.1021/acsami.4c11797)
Supplement: Supplementary file 1 — am4c11797_si_001.pdf [file am4c11797_si_001.pdf]

Supporting Information

Non-flammable ether and phosphate-based liquid electrolytes for sodium-ion batteries

Wessel W.A. van Ekeren,[a] Alexandre M. Pereira,[b] Marcelo Albuquerque,[b]*

Luciano T. Costa,[b] and Reza Younesi[a]

[a] Department of Chemistry-Ångström Laboratory, Uppsala University, SE-751 21, Uppsala, Sweden

[b] MolMod-CS, Physical Chemistry Department, Institute of Chemistry, Fluminense Federal University, Campus Valonguinho, Niterói-RJ, Brazil, CEP 24020-141

[c] Institute of Physics, Fluminense Federal University, Campus Praia Vermelha, Niterói-RJ, Brazil, CEP 24210-346

* Corresponding author: wessel.vanekeren@kemi.uu.se

Table S1 Chemical properties of solvents used in this study.

**Solvent**	**Mw (g/mol)**	**Density (g/cm^3)**	**Melting point (°C)**	**Boiling point (°C)**	**Viscosity (mPa · s)**	**Dielectric constant**
Diglyme	134.18	0.94	-64	162	1.09	7.3 [1]
Tetraglyme	222.28	1.009	-30	266	3.73	7.79 [1]
TEP	182.16	1.07	-57	215	1.6	13.01
TMP	140.08	1.205	-46	197	1.3	21.26 [2]

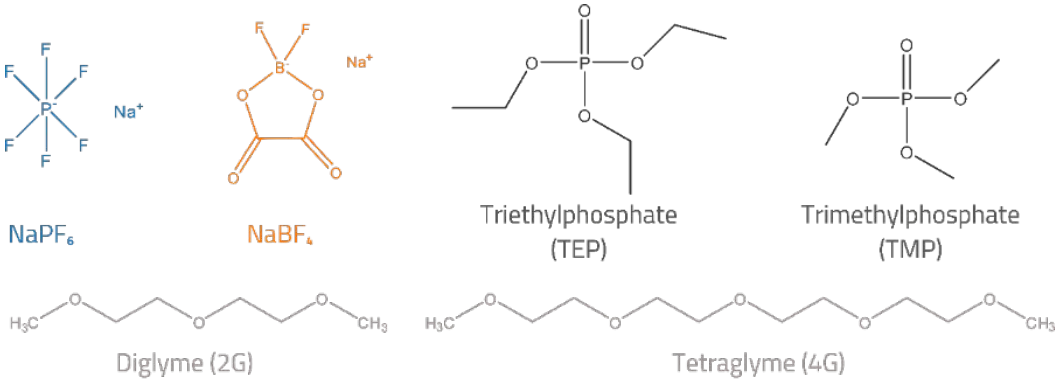

Figure S1 Overview of molecular structures of the chemicals used in this study.

Table S2 Overview of density and viscosity data at 20 C.

Electrolyte solution	T (°C)	Density (g/cm³)	Dynamic Viscosity (mPa s)
$NaBF_4$ G2:TEP (9:1)	20.00	1.117	2.060
$NaBF_4$ G2:TEP (7:3)	20.00	1.095	2.360
$NaBF_4$ G2:TEP (1:1)	20.00	1.073	3.097
$NaBF_4$ G2:TEP (3:7)	20.00	1.052	3.935
$NaBF_4$ G2:TEP (1:9)	20.00	1.030	4.538
$NaBF_4$ G2:TMP (9:1)	20.00	1.032	2.142
$NaBF_4$ G2:TMP (7:3)	20.00	1.081	2.855
$NaBF_4$ G2:TMP (1:1)	20.00	1.146	4.017
$NaBF_4$ G2:TMP (3:7)	20.00	1.194	4.319
$NaBF_4$ G2:TMP (1:9)	20.00	1.240	4.287
$NaBF_4$ G4:TEP (9:1)	20.00	1.125	7.440
$NaBF_4$ G4:TEP (7:3)	20.00	1.183	6.071
$NaBF_4$ G4:TEP (1:1)	20.00	1.108	5.942
$NaBF_4$ G4:TEP (3:7)	20.00	1.098	4.763
$NaBF_4$ G4:TEP (1:9)	20.00	1.088	4.258
$NaBF_4$ G4:TMP (9:1)	20.00	1.248	8.404
$NaBF_4$ G4:TMP (7:3)	20.00	1.217	7.716
$NaBF_4$ G4:TMP (1:1)	20.00	1.181	7.693
$NaBF_4$ G4:TMP (3:7)	20.00	1.140	5.157
$NaBF_4$ G4:TMP (1:9)	20.00	1.105	4.925
$NaPF_6$ G2:TEP (9:1)	20.00	1.158	3.121
$NaPF_6$ G2:TEP (7:3)	20.00	1.137	3.357
$NaPF_6$ G2:TEP (1:1)	20.00	1.113	3.756
$NaPF_6$ G2:TEP (3:7)	20.00	1.093	4.929
$NaPF_6$ G2:TEP (1:9)	20.00	1.067	5.341
$NaPF_6$ G2:TMP (9:1)	20.00	1.034	1.908
$NaPF_6$ G2:TMP (7:3)	20.00	1.081	2.226
$NaPF_6$ G2:TMP (1:1)	20.00	1.128	2.513
$NaPF_6$ G2:TMP (3:7)	20.00	1.255	2.859
$NaPF_6$ G2:TMP (1:9)	20.00	1.223	3.250
$NaPF_6$ G4:TEP (9:1)	20.00	1.071	6.526
$NaPF_6$ G4:TEP (7:3)	20.00	1.077	5.975
$NaPF_6$ G4:TEP (1:1)	20.00	1.087	5.106
$NaPF_6$ G4:TEP (3:7)	20.00	1.157	4.358
$NaPF_6$ G4:TEP (1:9)	20.00	1.107	3.166
$NaPF_6$ G4:TMP (9:1)	20.00	1.086	6.215
$NaPF_6$ G4:TMP (7:3)	20.00	1.123	5.692
$NaPF_6$ G4:TMP (1:1)	20.00	1.161	5.100
$NaPF_6$ G4:TMP (3:7)	20.00	1.197	4.431
$NaPF_6$ G4:TMP (1:9)	20.00	1.233	3.762

An overview of the dynamic viscosity values (measured at 20 °C) for the two salts $NaBF_4$ and $NaPF_6$ in various glymes:phosphate mixtures is shown in Fig. S2.

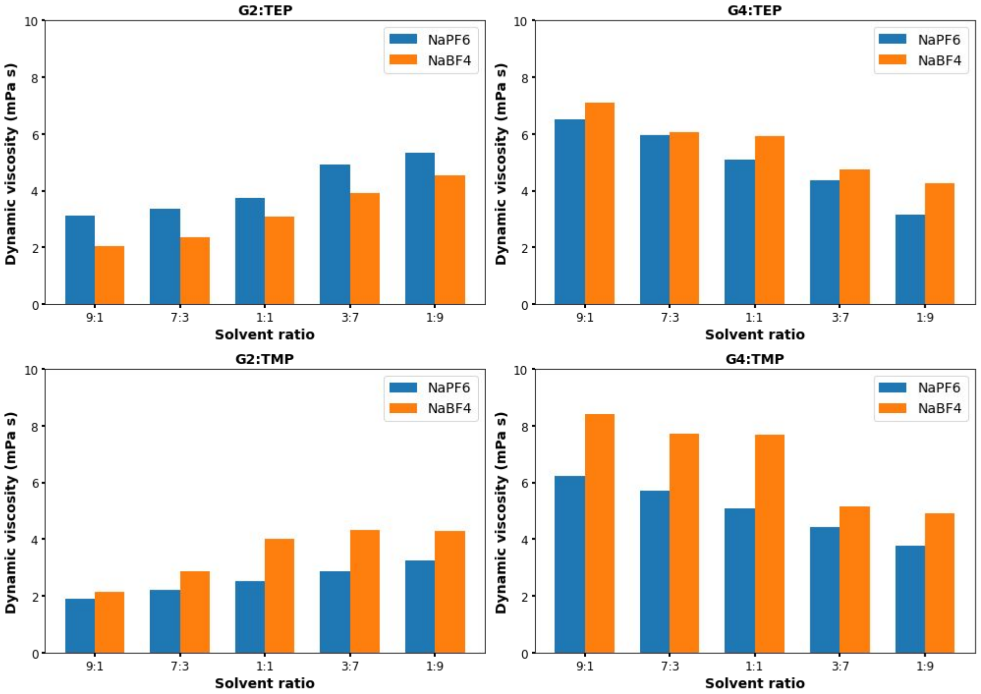

Figure S2 Dynamic viscosity data for 1.0 m salt in each specific glyme:phosphate solvent mixture at 20 °C.

Flammability test of 1.0 m $NaBF_4$ in tetraglyme is shown in Fig. S3.

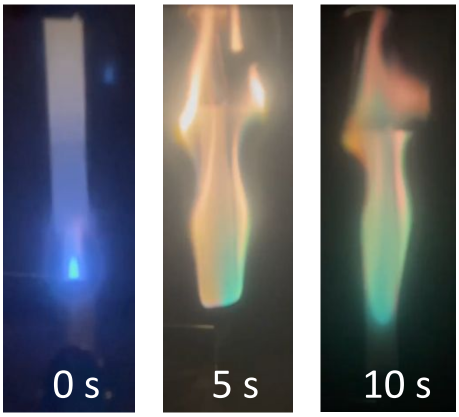

Figure S3 Flammability test of 1.0 m $NaBF_4$ in tetraglyme.

Effects of charges on the molecular dynamics equilibrated densities for the 2G:TMP (1:1 vol ratio) with $NaBF_4$ salt are shown in Fig. S4, which match well with the experimental studies.

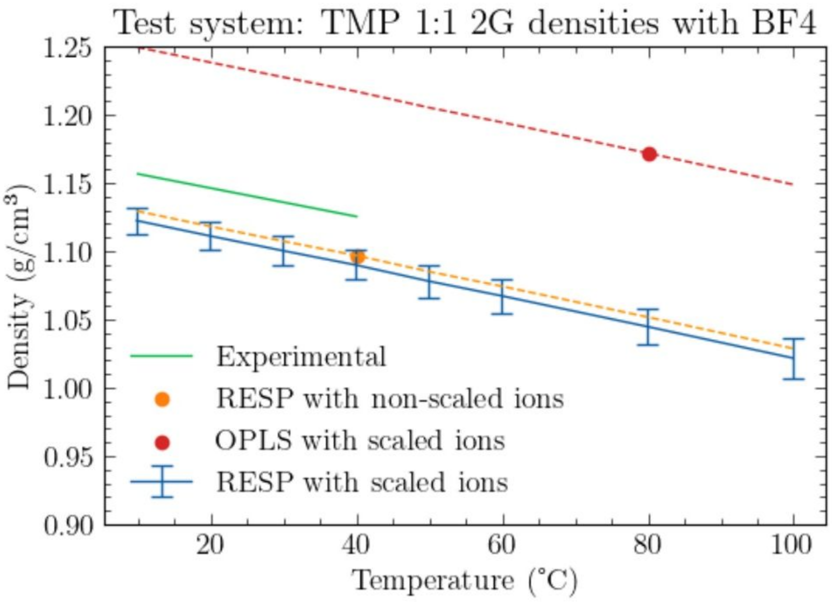

Figure S4 Effects of charges on MD equilibrated densities for the TMP 1:1 2G with BF_4^- ions.

An overview of the values of the HOMO (Highest Occupied Molecular Orbital) and LUMO (Lowest Unoccupied Molecular Orbital) orbitals energies for the 2G, 4G, TMP, and TEP molecules are shown in Fig. S5.

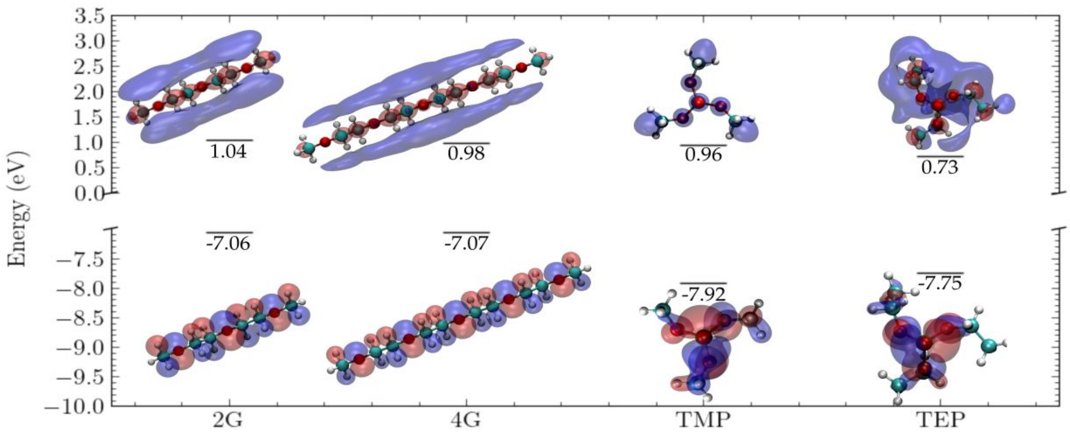

Figure S5 The HOMO-LUMO energy levels of the glyme (2G and 4G) and phosphate (TMP and TEP) solvents.

Computed electronegativity values, as shown in Table S3, confirm that glyme molecules are indeed less electronegative than alkyl phosphate molecules.

Table S3 HOMO and LUMO gap and calculated electronegativities for TMP, TEP, 2G, and 4G.

Molecule	HOMO-LUMO gap	Electronegativity
2G	-8.100800	3.007700
4G	-8.051100	3.043150
TMP	-8.870800	3.479600
TEP	-8.473000	3.509000

Table S4 Number of ions in each system, where Na^+ is equal to BF_4^- or PF_6^-.

Glyme:Phosphate	9:1 (450:50)	1:1 (250:250)	1:9 (50:450)
TMP:2G	35	32	30
TEP:2G	36	39	42
TMP:4G	52	42	31
TEP:4G	54	49	44

The molecular structure shown in Fig. S6 below illustrates the synergistic interaction between BF_4^- and TEP, suppressing the interaction of the anion with 2G.

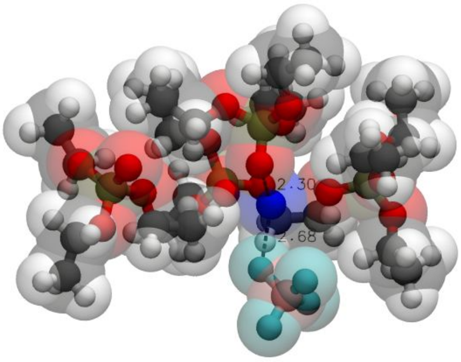

Figure S6 Molecular structure of the cluster formed by TEP, Na^+, and BF_4^-.

In Fig. S7 the galvanostatic cycling data is shown for the non-flammable solvent mixtures, showing a similar type of capacity fade as for the flame-retardant (7:3, 1:1 ratios) solvent mixtures.

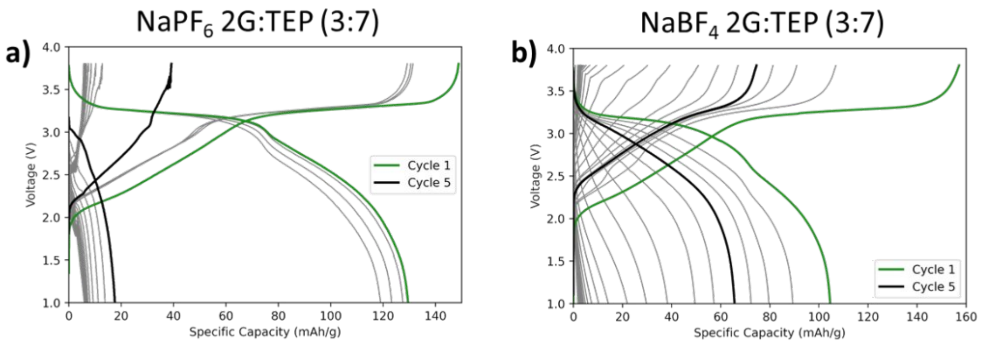

Figure S7 Cycling voltage profiles of $NaPF_6$ and $NaBF_4$ in non-flammable solvent mixtures.

References

1 S. Murov, Properties of Organic Solvents, http://murov.info/orgsolvents., (accessed 20 June 2024).

2 H. V. T. Nguyen, J. Kim and K. Lee, *J. Mater. Chem. A*, 2021, 20725–20736.
